# Hydroxyurea Treated β-Thalassemia Children Demonstrate a Shift in Metabolism Towards Healthy Pattern

**DOI:** 10.1038/s41598-018-33540-6

**Published:** 2018-10-11

**Authors:** Ayesha Iqbal, Saqib Hussain Ansari, Sadia Parveen, Ishtiaq Ahmad Khan, Amna Jabbar Siddiqui, Syed Ghulam Musharraf

**Affiliations:** 1 H.E.J. Research Institute of Chemistry, International Center for Chemical and Biological Sciences, University of Karachi, Karachi, 75270 Pakistan; 2 Dr. Panjwani Center for Molecular Medicine and Drug Research, International Center for Chemical and Biological Sciences, University of Karachi, Karachi, 75270 Pakistan; 3 Department of Pediatric Hematology & Molecular Medicine, National Institute of Blood Diseases and Bone Marrow Transplantation, Karachi, 75300 Pakistan

## Abstract

Augmentation of fetal hemoglobin (HbF) production has been an enduring therapeutic objective in β-thalassemia patients for which hydroxyurea (HU) has largely been the drug of choice and the most cost-effective approach. A serum metabolomics study on 40 patients with β-thalassemia prior to and after administration of HU was done along with healthy controls. Treated patients were divided further into non-responders (NR), partial (PR) and good (GR) per their response. 25 metabolites that were altered before HU therapy at p ≤ 0.05 and fold change >2.0 in β-thalassemia patients; started reverting towards healthy group after HU treatment. A prediction model based on another set of 70 HU treated patients showed a good separation of GR from untreated β-thalassemia patients with an overall accuracy of 76.37%. Metabolic pathway analysis revealed that various important pathways that were disturbed in β-thalassemia were reverted after treatment with HU and among them linoleic acid pathway was most impactfully improved in HU treated patients which is a precursor of important signaling molecules. In conclusion, this study indicates that HU is a good treatment option for β-thalassemia patients because in addition to reducing blood transfusion burden it also ameliorates disease complications by shifting body metabolism towards normal.

## Introduction

Fetal hemoglobin (HbF) is a type of hemoglobin tetramer that consist of two α and two γ chains. During the development of fetus it plays the main role as oxygen transporter^1^. Nearby birth, this HbF is substituted largely by HbA as a result of gene expression switching from γ to β (beta) globin chain^2^. However, infants suffering from β-thalassemia lack functional β globin due to loss of function mutations in *HBB* gene, fail to produce normal adult hemoglobin (HbA) and start showing signs and symptoms of the disease^3^. Imbalance in the α/β-chain ratio is a hallmark of β-thalassemia that can be reduced by compensating defaulted chain of the β-globin molecule with increased production of the γ-globin that ultimately form HbF^4^. This HbF induction limits the need for blood transfusion along with iron chelation to prevent complications related to iron overload by transfusion therapy^5,6^ and also diminishes need of potentially risky treatment option i.e. transplantation of allogenic bone marrow stem cells^7,8^.

Multiple γ-globin gene inducing therapeutic agents have been identified^9–13^. Among all these agents hydroxyurea (HU) is a Food and Drug Administration (FDA) approved HbF inducer^14^. A score of studies have identified that HU is well tolerated in clinical practice and can be implicated as an another option of β-thalassemia treatment^9,15–17^. HU is an antineoplastic agent used for myeloproliferative disorders. After being identified as a potent HbF inducer, HU became one of the key therapeutic agents for augmentation of HbF^9^ and it is also a cost effective approach as compared to other treatment options like bone marrow transplant and regular blood transfusion in conjugation with iron chelation costing US$3,000 per patient annually^18^. The most commonly accepted mechanism of HU is its cytotoxic effect resulting in stress erythropoiesis^19^. However, the exact mechanisms by which HU induces HbF production are not fully understood and is still controversial^20^. Also the response to HU therapy in β-thalassemia varies from patient to patient, some showed good respond to the therapy with hemoglobin maintained without any requirement of blood transfusions while some showed no difference in their hematological parameters^21,22^ as a result of which data on predictors of response remain inconsistent.

Currently, more than 200 causative molecular defects have been described so far in the β-globin gene causing β-thalassemia and also relation of some mutations have been found which respond to hydroxyurea^23–25^. Previously it was identified that the metabolite profile of β-thalassemia patients significantly differs from healthy^26^. Studies have outlined the changes in metabolite profile of leukemia patients^27^ and fatty acid profile of β-thalassemia patients^28^ prior and after recieving HU therapy. However, no study has addressed the relation of β-thalassemia patient’s metabolome and metabolic pathways to the HU treatment. Though, this area can produce interesting and significant insights regarding effects of HU treatment on various metabolic pathways as various studies of metabolomics in association to pathway analysis have provided noteworthy findings^29–32^ also in analyzing response and dose of drug^33^. Therefore, in this study we aimed to overcome this lacking by finding out the metabolomics pattern changes of β-thalassemia patients after receiving HU treatment and to recognize the differential metabolites among variable responders i.e. non-responders (NR), partial responders (PR) and good responders (GR) to HU treatment.

## Material and Method

### Patient’s Selection and Classification

The patients suffering with β-thalassemia for this study were selected in National Institute of Blood Disease and Bone Marrow Transplantation (NIBD), Karachi, Pakistan after the approval of the Institutional Review Board (IRB), ethic committee of the hospital (Approval # BTIHS-KHUT-001) as per The International Council for Harmonization of Technical Requirements for Pharmaceuticals for Human Use and Good clinical practice (ICH GCP) guidelines. Whereas the approval of experimental protocols was taken from the ethic committee of primary research institute (International Center for Chemical and Biological Sciences, ICCBS) i.e. Independent Ethic Committee (IEC) (Approval #-023-HB-2017). This research work has been done according to the ethical standards in the Declaration of Helsinki. Our study involved 40 β-thalassemia cases and their blood was collected before initiating and after receiving the treatment with HU and 70 samples of other patients treated with HU whose sample prior to treatment was not collected. Confirmed diagnosis of disease was performed by *HBB* gene mutation analysis. β-thalassemia mutations were identified by polymerase chain reaction-amplification refractory mutation system (PCR-ARMS). Patients received HU for the treatment at a dose range of 16–20 mg/kg/d and after receiving treatment for at least 6–12 months, these patients were further divided into three groups per their response i.e. non-responders (NR), partial responders (PR) and good responders (GR). Patients whose Hb maintained at >7 g/dl after receiving HU and without further blood transfusion were grouped as good responders, likewise partial responders were those who exhibited 50% reduction while non-responders exhibited <50% reduction in the need of blood transfusion post treatment with HU. Volunteers for healthy sample collection were also recruited at Dr. Panjwani Center for Molecular Medicine and Drug Research (PCMD).

### Inclusion and Exclusion Criteria of Patients

For starting HU therapy following inclusion was followed: Patient have been registered at NIBD, diagnosed as transfusion dependent β-thalassemia with Hb less than 7 g/dL, absence of or very low HbA amount with high HbF and blood transfusions >8/year, blood was collected before blood transfusion in case if needed along with fasting of 4–8 hours. Also, patients did not receive any therapy other than HU (at each collection other therapies were hold 15 days prior to sampling). Exclusion criteria included: Patients with HU hypersensitivity, any β-thalassemia unrelated other chronic illness evidence and unwillingness to enroll in the study or initiating therapy. Informed consent in written form was attained from all the participants involved in this work including healthy controls as well as patients or from their guardians.

### Sample and Data Collection from Participants

Collection of blood samples was done according to the ethical standards as laid down in the declaration of Helsinki. First sample was collected before starting HU therapy while second or treated sample was collected from patients after receiving 6–12 months of HU treatment in fasting. Near 5 cc of venous blood was drawn from each of the participant by venipuncture and collected in gel-based BD vacutainer tubes (BD Franklin Lakes NJ, USA, REF: 367381). Serum separation was achieved by centrifugation at 2000 rpm for 10 minutes at 4 °C; aliquoted and stored immediately at −80 °C till further processing of sample. A complete and thorough questionnaire was also filled from all the patients. Patients and healthy controls basic details are presented in Table S1.

### Reagents and Solvents

Solvents of analytical grade were used for gas chromatography-mass spectrometric (GC-MS) analysis. Reagents and solvents included methanol, hexane (Tedia, Tediaway, Fairfield, USA), myristic-d_27_ acid, *N,O*-bis(trimethylsilyl) trifluoroacetamide (BSTFA) with trimethylchlorosilane (TMCS) (Sigma-Aldrich, St. Louis, Missouri, USA), methoxylamine hydrochloric (Acros Organic, New Jersey, USA), Pyridine (Lab-Scan, Bangkok, Thailand) and Mill-Q/deionized water (Millipore, Billerica, MA, USA).

### Sample Preparation, Derivatization and GC-MS Analysis

Details of protocol that is followed in our study for metabolomics sample preparation has been reported previously reported^34^. Shortly, first proteins were precipitated by adding 800 µL of chilled methanol in 100 µL serum containing 20 µL of myristic acid (2 mg/mL) as internal standard. Resulting supernatant was further subjected to solid phase extraction (SPE) using a 96 well plate (Strata C18-E, 55 µm pore size, 70 Å particle, 100 mg sorbent/1 mL Phenomenex, USA) under vacuum (AHC-7502, Phenomenex, USA). After sample loading, the phase was washed with 300 µL of water and metabolites were eluted with 600 µL methanol. The eluent was finally dried in vacuum at room temperature and stored at 4 °C until analysis. Derivatization of dried samples was performed by addition of 50 µL methoxylamine hydrochloride (15 µg/ µL in pyridine) followed by addition of 50 µL BSTFA with 1% trimethylchlorosilane for formation of trimethylsilyl derivatives. Then samples were analyzed on GC-MS with minor modifications in previous method^35^. Initially the oven temperature was fixed at 50 °C for 1 min then temperature was raised in three steps. In first step temperature raised at a rate of 10 °C/min to 80 °C for 3 min then again 10 °C/min to 180 °C for 3 min and in final step 15 °C per min raise to 300 °C for 5 min. After maintaining the temperature at 300 °C for five minutes, a post run was applied by further increasing to 305 °C for 1 minute. Retention time was locked to the internal standard at 20.070 min. Electron impact ionization (EI) was ionization source for the GC-MS analysis at 70 eV. Data acquisition was done in full scan mode from 50–650 *m/z* in 0.5 seconds scan time. To limit the chances of cross contamination a blank was run in-between samples. Mass calibration was done with perfluorotributylamine (PFTBA).

### GC-MS Data Pre-processing and Statistical Analysis

Agilent Mass Hunter Qualitative Analysis software (version B.04.00) was used for data processing. Parameters applied for peak integration and deconvolution parameters have been described previously^34,35^. The GC-MS spectra were uploaded on Mass Profiler Professional (MPP) software 12.5 for multistep statistical processing of data as previously reported^34^. Statistical significance analysis was done using T-Test Unpaired for comparing 5 groups involved in study i.e. healthy as control group, HU untreated β-thalassemia patients and HU treated patients group which was further classified into GR, PR and NR. Using T-Test Unpaired and Benjamini Hochberg FDR as multiple testing correction, the fold change (FC) of 2.0 was applied to compare these groups. Tukey’s Honest Significant Difference (HSD) post Hoc test was then applied to identify metabolites that were accountable for significant differences and also that were common in these five groups. Hierarchical clustering was also executed by applying Pearson’s uncentered distance metric and complete linkage. A partial least squares discriminant analysis (PLS-DA) model was built for healthy versus HU treated and HU untreated β-thalassemia patients. Pathway analysis of altered metabolites was also carried out using Metaboanalyst 3.0 (www.metaboanalyst.ca/)^36^ which is a web based software linked to huge databases such as KEGG (Kyoto Encyclopedia of Genes and Genomes) (http://www.genome.jp/kegg/)^37–39^ and HMDB (Human Metabolome Database) (http://www.hmdb.ca/).

## Results

Serum metabolite profile of 40 β-thalassemia patients before HU therapy was compared with their metabolite profile after treatment, in addition 70 other samples of HU treated β-thalassemia patients and 60 samples of healthy volunteers were used as controls. In total 210 serum samples were analyzed by using GC-EI-MS. Statistical analysis and multivariate data investigation including hierarchical clustering, Tukey HSD post-hoc analysis and PLS-DA plot were carried out to detect comparative alteration of statistically significant metabolites in-between healthy and β-thalassemia patient samples prior to and after getting treatment with HU including further three groups i.e. NR (non-responder), PR (partial responder) and GR (good responder).

### Significance Testing and Fold Change

**Figure 1:**
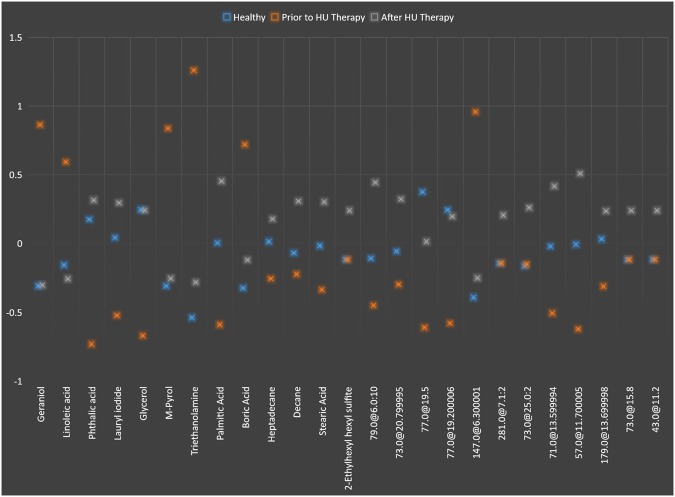
Pattern of the significantly differentiated 25 metabolites at FC > 2 in β-thalassemia patients before and after HU therapy along with healthy group as control, plotted on average intensity values of each metabolite.

**Figure 2:**
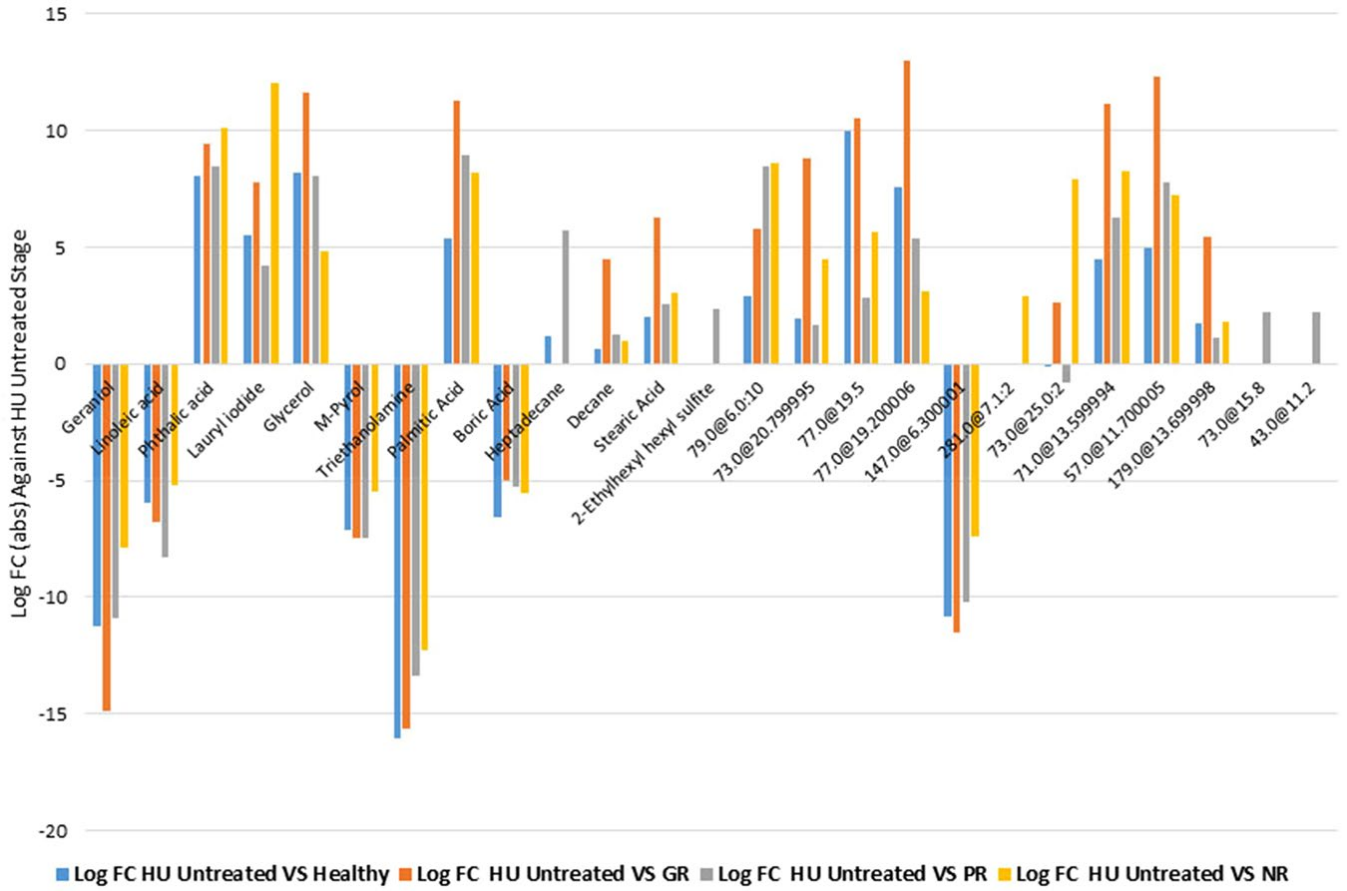
Plot of log FC (fold change) of each significantly altered metabolite in GR, PR, NR and healthy group against their sample before treatment with HU.

On total of 717 metabolites, significant testing with fold change analysis was carried out in this experiment. At the level of p ≤ 0.05 and FC > 2, a list of 25 compounds was produced in which some were up and down-regulated in β-thalassemia patients in comparison to other 4 groups. Briefly, 11, 8, 10 and 11 metabolites were down regulated while, 14, 17, 15 and 14 were up regulated in GR, PR, NR and healthy, respectively in comparison to their sample prior to HU treatment. Out of twenty-five thirteen metabolites; geraniol, linoleic acid, phthalic acid, lauryl iodide, glycerol, M-pyrol, triethanolamine, palmitic acid, boric acid, heptadecane, decane, stearic acid and 2-ethylhexyl hexyl sulfite; were recognized putatively by relating the mass spectral peaks with the spectral data available in the National Institute of Standards and Technology (NIST) mass spectral (Wiley registry NIST 11) library at similarity index parameter of ≥70%. The difference in the pattern of significantly differentiated metabolites in β-thalassemia patients post receiving HU treatment can be seen in Fig. 1 plotted on the average intensities of these 25 metabolites. In this figure, it was quite evident that after HU therapy metabolite pattern of same β-thalassemia patients inclined near the healthy controls. While the plot of log FC of significantly altered metabolites in all groups is presented separately in Fig. 2 indicating that most of these metabolites have changed sufficiently in all groups of our study as compared to HU untreated samples while two identified metabolites i.e. heptadecane and 2-ethylhexyl hexyl sulfite differ in one or two groups. The list of metabolites is represented in table in which identified one are with their name and CAS (Chemical Abstracts Service) registry numbers along with fold change while base peak and retention time (RT) are provided for unidentified one in Supplementary Table S2. The mass spectra of twelve unidentified metabolites are also provided in supplementary data as Fig. S1.

Tukey’s HSD post Hoc test was also applied to find significantly expressed metabolites among groups and its summary is shown in Table 1. Nine metabolites significantly differentiate healthy from β-thalassemia but in case of good response after treatment with HU their difference of serum metabolites reduced to three. GR group showed more dissimilar metabolites with their sample prior to HU treatment i.e. eighteen out of 25 metabolites were dissimilar. Also, the GR group was least discriminable from the healthy and 22 metabolites were common in healthy and GR patients. Which indicates that metabolic profile of GR was most significantly reverted to normal in β-thalassemia patients. But between NR and PR group less difference of metabolite is found with their sample prior to treatment with HU i.e. 13 and 15 respectively. Seven metabolites out of 25 were also similar among NR and HU untreated samples, therefore the metabolite profile of these patients also shows that metabolism of these were less reverted to normal. The difference of metabolites was also found between sub groups of patients receiving HU treatment i.e. GR, PR and NR. 4 metabolites differ between GR and NR out of which 2 were identified as geraniol and decane. However, PR varied from GR with 7 metabolites and out of which 2 metabolites were identified i.e. heptadecane and sulfurous acid while remaining were unidentified.

**Table 1:** Summary of Tukey HSD post hoc test representing differentiated and common metabolites among hydroxyurea (HU) untreated, healthy controls, good responders (GR), partial responders (PR) and non-responders (NR).

Group Name	GR	Untreated	Healthy	PR	NR
GR	25	18*	3*	7*	4*
Untreated	7**	25	9*	15*	13*
Healthy	22**	16**	25	4*	2*
PR	18**	10**	21**	25	7*
NR	21**	12**	23**	18**	25

* represent the differentially expressed metabolites between the groups.

** represents the undifferentiated metabolites among groups.

### Cluster Analysis

**Figure 3:**
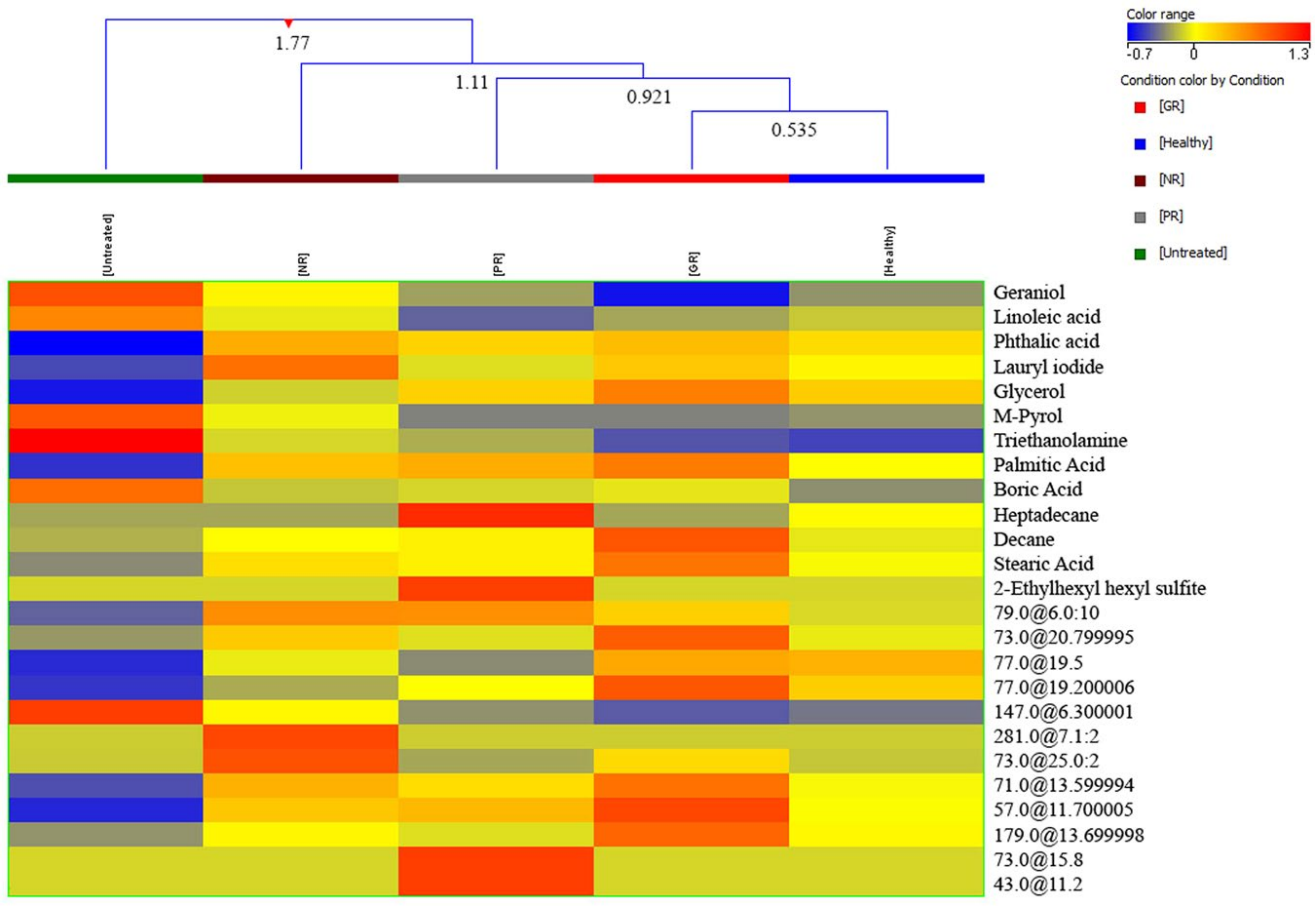
Hierarchical clustering dendogram showing comparison of five groups of samples i.e. healthy controls, β-thalassemia patients without HU treatment, GR (good responders), PR (partial responders) and NR (non-responders) to HU therapy. Note that GR group is least dissimilar to healthy group.

Hierarchical clustering was done using average intensities of twenty-five differentiating metabolites. The five groups were clustered into four classes (Fig. 3). GR and healthy were clustered together in class I with least dissimilarity of 0.53, while three groups including PR, GR and healthy samples were clustered together in class II with a dissimilarity level of 0.921. And in class III four groups i.e. NR, PR, GR and healthy samples were at dissimilarity level of 1.11. All the five groups (healthy controls, HU untreated, GR, PR and NR patients) were clustered in class IV revealing a highest dissimilarity level of 1.77 indicating that metabolite profile of patients before receiving HU was most dissimilar from the healthy and other three groups of HU treated patients. In our samples, the most common mutation was intervening sequence (IVS)1–5 and better response to HU has been correlated with it so we have also produced a Venn diagram to compare metabolites of IVS1–5 with other mutations after treatment (Fig. S2). In which 5 and 16 are the number of metabolites shared by IVS1–5 and other mutations that were down- and up-regulated as compared to healthy individuals respectively. While 7, 1 and 1,7 metabolites were differently up and down regulated in IVS1–5 and other type of mutations correspondingly.

### Class Prediction Model

**Figure 4:**
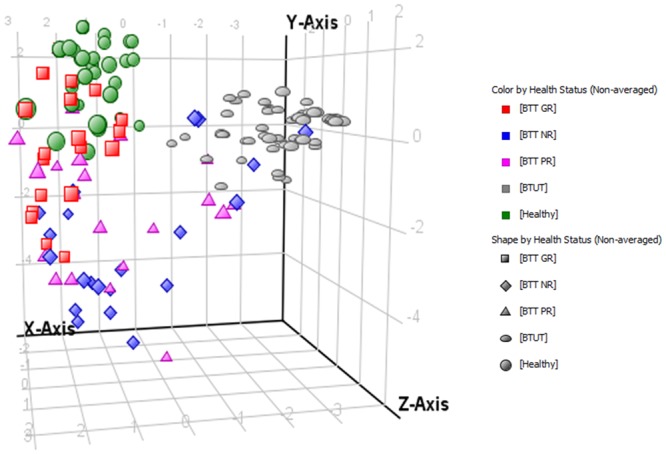
3D Partial Least Square-Discriminant Analysis (PLSDA) score plot based on the differentiating metabolite profile data of serum of five groups showing a noteworthy separation trend between the GR and healthy to HU untreated and NR group while PR was the most dispersed group.

A prediction model using another set of 70 samples of HU treated group along with healthy and HU untreated group was built by multivariate data analysis. This model included 25 GR, 23 PR, 22 NR, 64 HU untreated and healthy samples. Based on statistically differentiating as well as important metabolites that also have difference in expression between these five clusters, samples were grouped into distinct classes by supervised partial least squares discriminant analysis (PLS-DA). Plots of PLS-DA scores are given in Fig. 4 showing a noteworthy separation trend between the GR and healthy to HU untreated and NR group. In PLS-DA some of the GR samples were predicted as healthy which is a good sign. Therefore, the profile of GR to HU treatment indicates that HU not only diminished the need of blood transfusion but also shifts their body metabolism towards normal by which it reduces the other complications of β-thalassemia. Most dispersed group was PR and these samples were also least correctly predicted. As it can be observed in Table 2 that PR samples were predicted in all groups including healthy, GR, PR, NR and HU untreated patients. So, the diversity in metabolite pattern of this groups may also be responsible for less response of these patients to HU therapy or vice versa. While also some NR samples were anticipated as HU untreated samples as a result of which overall accuracy of PLS-DA model was reduced to 76.37%. The details of prediction accuracy of all five groups of our study are mentioned in Table 2.

**Table 2:** Results of PLS-DA prediction model generated in the form of confusion matrix consisting of HU untreated β-thalassemia patients (n = 64), HU treated three groups of β-thalassemia patients i.e. GR (n = 25), PR (n = 23), NR (n = 22) and healthy controls (n = 61).

	ΒTT GR Predicted	BTT NR Predicted	BTT PR Predicted	BTUT Predicted	Healthy Predicted	Accuracy
True BTT GR	17	0	03	00	05	68.000
True BTT NR	00	15	02	05	00	68.182
True BTT PR	05	09	03	02	04	13.043
True BTUT	00	00	00	64	00	100.000
True Healthy	07	00	01	00	40	83.333
Overall Accuracy						76.374

BTT (β-Thalassemia Treated with Hydroxyurea), GR (Good Responders), PR (Partial Responders), NR (Non-esponders) & BTUT (β-Thalassemia Untreated with Hydroxyurea).

### Pathway Analysis

**Figure 5:**
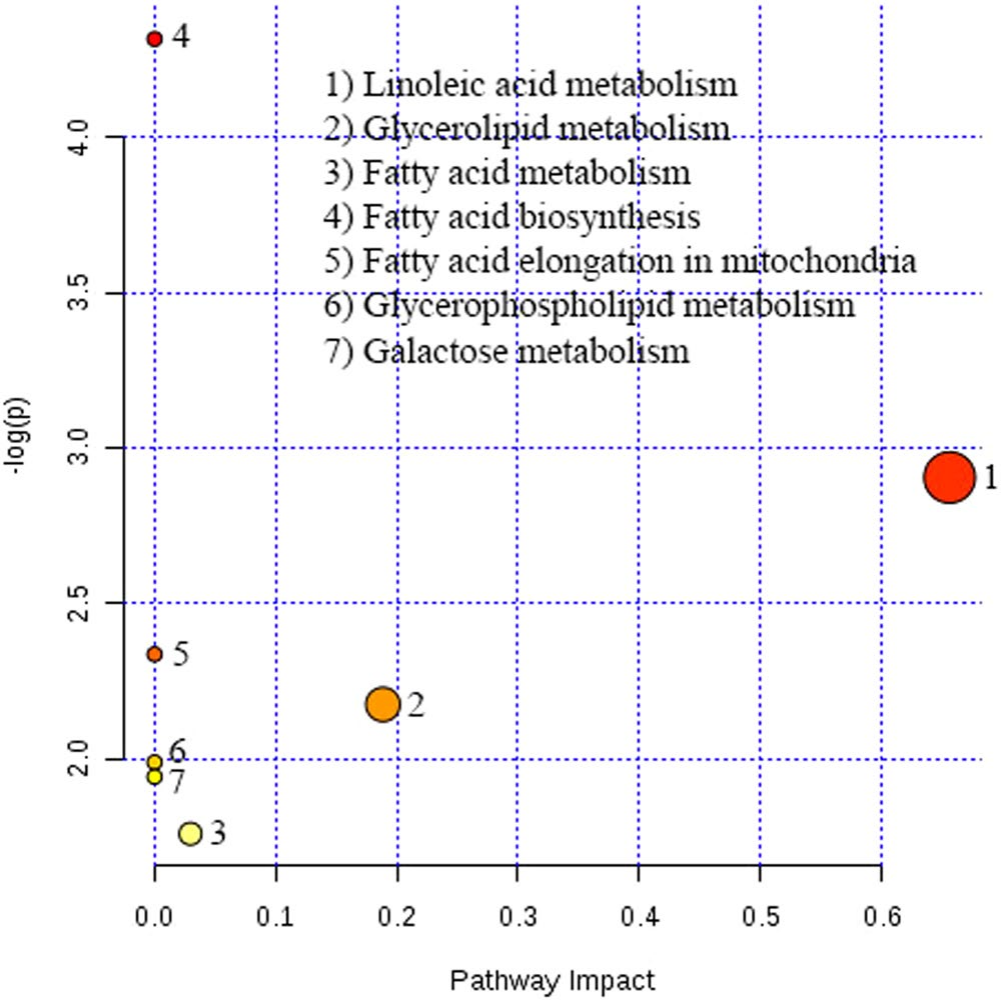
Summary of pathway analysis of metabolites that were dysregulated in β-thalassemia patients before treatment with HU as compared to healthy controls and after treatment with HU.

To identify disturbed pathways of metabolism attributable to altered metabolites in serum of four groups in comparison to β-thalassemia samples before HU treatment. The pathway analysis summary formed on the basis of dysregulated metabolites are shown in Fig. 5. While the pictures of distinctive identified pathways are provided in supplementary information i.e. Fig. S3. Out of thirteen identified metabolites five metabolites were found to be involved in metabolism pathways that were distressed in individuals with β-thalassemia before recieving HU treatment and these include linoleic acid, glycerol, triethanolamine, stearic acid and palmitic acid. The total number of pathways found to be altered and then reverted after HU therapy is seven which are listed in supplementary information (Table S3).

## Discussion

In this study we have compared the metabolites pattern of β-thalassemia patients prior to and after receiving treatment with HU for 6–12 months to induce HbF. Overall, most of the metabolites in serum of HU treated patients have quite similar pattern of regulation but difference was found in their intensities as compared to their sample before treatment with HU. The possible role of metabolites having common pattern in all HU treated groups can be defined by consulting to the HMDB^40–42^. Linoleic acid is required in the biosynthesis of prostaglandins and cell membranes and its abnormal levels have been found in cirrhosis^43^, hypertension^44^ and cancers^45^. In β-thalassemia samples it is most possibly increased due to inflammation, oxidative damage and increased red blood cell (RBC) membrane turn over, but its level reduced to normal after HU treatment in all types of responders as these complications started resolving. Palmitic and stearic acids are involved in multiple metabolic pathways such as plasmalogen synthesis, β-oxidation of fatty acids, bile acid and fatty acid biosynthesis etc. These two fatty acids are also important for maintaining dynamics of cell membranes and their decrease in body further result in reduced strength of RBCs membrane, as HU decreases RBCs degradation leading to improve membrane integrity therefore the levels of these two fatty acids are also returned to normal post treatment with HU. Glycerol molecules can be converted into glucose by liver to meet the body’s energy requirements. Thus, it is logical that after treatment with HU the metabolic stress is reduced in β-thalassemia patients so less glycerol is consumed for energy production and its serum levels went back to normal. Geraniol possesses anti-oxidant with anti-inflammatory activity and in healthy and HU treated samples it is decreased as compared to the HU untreated ones. Although it is an exogenous metabolite but due to oxidative stress in β-thalassemia its uptake increased in blood. Because HU treatment reduces oxidative stress and inflammation^46^ therefore geraniol levels were more in β-thalassemia before treatment as compared to treated group to combat oxidative and inflammatory damage in disease. Altered levels of M-pyrol are seen in urinary tract infections, common complication of β-thalassemia because of predisposing factors. HU therapy reduces these predisposing factors, so all HU treated β-thalassemia without regarding to their response have M-pyrol levels closer to healthy individuals. Decane and lauryl iodide are found in cell membranes and their abnormal concentrations have been found in fatty liver diseases and infections. We have found that HU treated patient’s metabolome pattern of these two metabolites was like healthy persons. Heptadecane belongs to the family of acyclic alkanes and is found in membranes of cell. It is one of the metabolite whose levels were different among healthy and GR along with NR however similar in PR.

In our pathway analysis, linoleic acid, glycerolipid and fatty acid metabolism were most affected whereas metabolites blamable for these abnormalities are linoleic acid, glycerol and palmitic acid respectively. Glycerol and palmitic acid are involved in more than one pathway, so it indicates that alteration of these two metabolites caused changes in galactose metabolism, fatty acid biosynthesis and elongation in mitochondria, respectively in addition to above mentioned pathways. Although in anemia the pathways which are most commonly affected include glycolysis, the oxidative pentose phosphate pathway (OPPP), the glutathione cycle, nucleotide metabolism and MetHb reductase^47^ but we didn’t found any of these pathway directly involved in causing pathogenesis of β-thalassemia in our study. So, it will be reasonable to state that the pathways altered are significantly involved in complicating β-thalassemia and are not related to other types of anemias.

Linoleic acid pathway was most impact fully improved in HU treated patients. It is an essential poly unsaturated fatty acid (PUFA) for humans as it is not synthesized in body and plays important role by producing hormone like lipids that act as signalling molecules and induce inflammation and blood clotting. Although, altered levels of linoleic acid have been reported in multiple disorders, but it is correlated mostly with cardiovascular diseases. As β-thalassemia patients suffer from iron overload so in these patients linoleic acid metabolism was increased as a cardio protective agent. In addition, increase RBCs production and increased inflammatory response has been associated with transfusion dependent β-thalassemia patients. Therefore, these two factors are also likely to be responsible for elevated metabolic pathway of linoleic acid as it produces inflammatory mediators and to overcome RBC degradation. But when HU was given to these patients it ameliorated all three complications therefore linoleic acid metabolism also revert to normal as in healthy individuals. Glycerolipid and fatty acid metabolism was also augmented for compensating need of fatty acids but when this need is subsided by HU treatment, so these pathways also return to normal level. Comprehensively we can state that metabolic stress reduces in patients with β-thalassemia after receiving HU therapy which consequently result in normal metabolism of various altered pathways.

## Conclusion

In conclusion, this study showed that hydroxyurea therapy in patients with β-thalassemia restores disturbed metabolic profile to normal in addition to its reported reduction in need for blood transfusion. Our study established that serum metabolite profiling by using GC-EI-MS can be used for the establishment of a profile that can indicate weather hydroxyurea in β-thalassemia patients is effective and it should be continued or not which means that the metabolism is reverting towards normal or not because not only good responders, but also partial and somewhat non-responders showed inclined metabolic pattern towards healthy. Furthermore, our study is the first one to identify and describe metabolomic differences in the serum of healthy individuals and patients with β-thalassemia before and after receiving hydroxyurea an understanding that can be used to assess disease prognosis and response to treatment as well as supporting that hydroxyurea is a good choice for β-thalassemia sufferers.

## Electronic supplementary material

Supplementary Information
