# Supplementary material for: Hydroxyurea Treated β-Thalassemia Children Demonstrate a Shift in Metabolism Towards Healthy Pattern
Source: Sci Rep. 2018 Oct 11;8:15152. doi: 10.1038/s41598-018-33540-6 (PMC6182004; doi:10.1038/s41598-018-33540-6)
Supplement: Supplementary file 1 — Supplementary Information [file 41598_2018_33540_MOESM1_ESM.pdf]

Hydroxyurea Treated β-Thalassemia Children Demonstrate a Shift in Metabolism Towards Healthy Pattern

Ayesha Iqbal,[b] Saqib Hussain Ansari,[c] Sadia Parveen,[c] Ishtiaq Ahmad Khan,[b] Amna Jabbar Siddiqui [a] and Syed Ghulam Musharraf [a,b *]

[a] *H.E.J. Research Institute of Chemistry, International Center for Chemical and Biological Sciences, University of Karachi, Karachi-75270, Pakistan*

[b] *Dr. Panjwani Center for Molecular Medicine and Drug Research, International Center for Chemical and Biological Sciences, University of Karachi, Karachi-75270, Pakistan*

[c] *Department of Pediatric Hematology & Molecular Medicine, National Institute of Blood Diseases and Bone Marrow Transplantation, Karachi-75300, Pakistan*

* Corresponding author. Tel.: +92 21 34824924-5; 34819010; fax: + 92 21 34819018-9.
E-mail address: musharraf1977@yahoo.com

Table S1: Age, weight and height of healthy normal subjects and β-thalassemia patients

S. No.	Parameter	β-Thalassemia Patients on HU	Healthy Volunteers
1.	Age (years)	9.93 ± 6.68	18.73 ± 7.93
2.	Weight (Kgs)	23.53 ± 12.78	47.375 ± 16.34
3.	Height (cms)	118.02 ± 31.79	156.5 ± 18.73

Table S2: List of altered metabolites in β-thalassemia patients prior treatment to HU in comparison to GR (good responders), PR (partial responders), NR (non-responders) and healthy controls

S. No.	Compound (CAS No.)	p (Corr) ([HU Untreated] Vs [Response])	Log FC (abs) ([HU Untreated] Vs [Response])	Regulation
**Differential Metabolites Between HU Untreated and GR**				
1.	Geraniol (106-24-1)	3.84E-09	-14.857795	down
2.	Linoleic acid (60-33-3)	0.42972735	-6.765212	down
3.	Phthalic acid (117-81-7)	0.002268163	9.410982	up
4.	Lauryl iodide (4292-19-7)	0.22254048	7.7674904	up
5.	Glycerol (56-81-5)	0.006577265	11.625441	up
6.	M-Pyrol (872-50-4)	0.15456924	-7.476566	down
7.	Triethanolamine (20836-42-4)	2.76E-07	-15.647676	down
8.	Palmitic Acid (57-10-3)	9.75E-06	11.249282	up
9.	Boric Acid (4325-85-3)	0.5134135	-4.9978504	down
10.	Heptadecane (629-78-7)	1	0	down
11.	Decane (124-18-5)	0.057890896	4.4753194	up
12.	Stearic Acid (57-11-4)	0.011514486	6.2951193	up
13.	2-Ethylhexyl hexyl sulfite (999397-36-5)	1	0	down
14.	79.0@6.0:10	0.07663433	5.811474	up
15.	73.0@20.799995	0.012075663	8.811704	up
16.	77.0@19.5	1.75E-04	10.53213	up
17.	77.0@19.200006	2.76E-07	12.994391	up
18.	147.0@6.300001	0.011514486	-11.498218	down
19.	281.0@7.1:2	1	0	down
20.	73.0@25.0:2	0.9648862	2.648282	up
21.	71.0@13.599994	0.001974755	11.140659	up
22.	57.0@11.700005	3.84E-09	12.321699	up
23.	179.0@13.699998	0.011514486	5.436824	up
24.	73.0@15.8	1	0	down
25.	43.0@11.2	1	0	down
**Differential Metabolites Between HU Untreated and PR**				
1.	Geraniol (106-24-1)	5.23E-05	-10.9257345	down
2.	Linoleic acid (60-33-3)	0.09600602	-8.323874	down
3.	Phthalic acid (117-81-7)	0.006918071	8.445266	up
4.	Lauryl iodide (4292-19-7)	0.64986116	4.220274	up
5.	Glycerol (56-81-5)	0.16835898	8.045367	up
6.	M-Pyrol (872-50-4)	0.09600602	-7.476566	down
7.	Triethanolamine (20836-42-4)	4.55E-05	-13.364202	down
8.	Palmitic Acid (57-10-3)	5.63E-04	8.970922	up

9.	Boric Acid (4325-85-3)	0.54504794	-5.2985153	down
10.	Heptadecane (629-78-7)	0.015582263	5.710911	up
11.	Decane (124-18-5)	0.6166823	1.2205789	up
12.	Stearic Acid (57-11-4)	0.36227182	2.5257506	up
13.	2-Ethylhexyl hexyl sulfite (999397-36-5)	0.36227182	2.358709	up
14.	79.0@6.0:10	0.005951495	8.493138	up
15.	73.0@20.799995	0.9264361	1.6715981	up
16.	77.0@19.5	0.36227182	2.816701	up
17.	77.0@19.200006	0.056861196	5.356953	up
18.	147.0@6.300001	0.031234756	-10.218083	down
19.	281.0@7.1:2	1	0	down
20.	73.0@25.0:2	1	-0.8061919	down
21.	71.0@13.599994	0.21747504	6.267552	up
22.	57.0@11.700005	5.63E-04	7.7781396	up
23.	179.0@13.699998	0.6166823	1.1058395	up
24.	73.0@15.8	0.36227182	2.210301	up
25.	43.0@11.2	0.36227182	2.1954303	up
**Differential Metabolites Between HU Untreated and NR**				
1.	Geraniol (106-24-1)	0.003548652	-7.859374	down
2.	Linoleic acid (60-33-3)	0.4903073	-5.2237744	down
3.	Phthalic acid (117-81-7)	4.60E-04	10.0987835	up
4.	Lauryl iodide (4292-19-7)	6.14E-04	12.017643	up
5.	Glycerol (56-81-5)	0.64721316	4.830533	up
6.	M-Pyrol (872-50-4)	0.42175508	-5.470641	down
7.	Triethanolamine (20836-42-4)	1.85E-04	-12.289695	down
8.	Palmitic Acid (57-10-3)	0.001518828	8.176298	up
9.	Boric Acid (4325-85-3)	0.40651155	-5.5626535	down
10.	Heptadecane (629-78-7)	1	0	down
11.	Decane (124-18-5)	0.6619495	0.9879475	up
12.	Stearic Acid (57-11-4)	0.31296006	3.0551002	up
13.	2-Ethylhexyl hexyl sulfite (999397-36-5)	1	0	down
14.	79.0@6.0:10	0.002770478	8.569718	up
15.	73.0@20.799995	0.4515015	4.4505167	up
16.	77.0@19.5	0.057112515	5.624808	up
17.	77.0@19.200006	0.31296006	3.1140907	up
18.	147.0@6.300001	0.25082156	-7.4067016	down
19.	281.0@7.1:2	0.31296006	2.920895	up
20.	73.0@25.0:2	0.037905566	7.9159417	up
21.	71.0@13.599994	0.031968586	8.260789	up
22.	57.0@11.700005	0.001518828	7.2176046	up
23.	179.0@13.699998	0.48273355	1.7867054	up
24.	73.0@15.8	1	0	down
25.	43.0@11.2	1	0	down

Differential Metabolites Between HU Untreated and Healthy Controls				
1.	Geraniol (106-24-1)	7.06E-08	-11.26246	down
2.	Linoleic acid (60-33-3)	0.017982906	-5.9744945	down
3.	Phthalic acid (117-81-7)	2.47E-04	8.073167	up
4.	Lauryl iodide (4292-19-7)	0.097781695	5.5243683	up
5.	Glycerol (56-81-5)	7.68E-04	8.204548	up
6.	M-Pyrol (872-50-4)	2.82E-05	-7.1544094	down
7.	Triethanolamine (20836-42-4)	7.10E-23	-16.062122	down
8.	Palmitic Acid (57-10-3)	0.01400611	5.389105	up
9.	Boric Acid (4325-85-3)	1.45E-04	-6.5730605	down
10.	Heptadecane (629-78-7)	0.4680696	1.180193	up
11.	Decane (124-18-5)	0.56389797	0.64677817	up
12.	Stearic Acid (57-11-4)	0.37845907	1.9860518	up
13.	2-Ethylhexyl hexyl sulfite (999397-36-5)	1	0	down
14.	79.0@6.0:10	0.31629103	2.9215834	up
15.	73.0@20.799995	0.4680696	1.933824	up
16.	77.0@19.5	8.04E-05	9.971461	up
17.	77.0@19.200006	0.001101988	7.5901365	up
18.	147.0@6.300001	8.49E-09	-10.843138	down
19.	281.0@7.1:2	1	0	down
20.	73.0@25.0:2	1	-0.09501304	down
21.	71.0@13.599994	0.16142486	4.508538	up
22.	57.0@11.700005	0.010302765	4.9603453	up
23.	179.0@13.699998	0.37845907	1.7412099	up
24.	73.0@15.8	1	0	down
25.	43.0@11.2	1	0	down

Table S3: List of dysregulated pathways in β-thalassemia patients before and after treatment with HU and in comparison to healthy controls.

Metabolic Pathway Name	Total Metabolites Present in Pathway	Hits	Raw p	-log(p)	Holm p	Impact
Fatty acid biosynthesis	49	2	1.33 E-02	4.32 E+00	1	0.00
Linoleic acid metabolism	15	1	5.48 E-02	2.90 E+00	1	0.66
Fatty acid elongation in mitochondria	27	1	9.67 E-02	2.34 E+00	1	0.00
Glycerolipid metabolism	32	1	1.14 E-01	2.17 E+00	1	0.19
Glycerophospholipid metabolism	39	1	1.37 E-01	1.99 E+00	1	0.00
Galactose metabolism	41	1	1.43 E-01	1.94 E+00	1	0.00
Fatty acid metabolism	50	1	1.72 E-01	1.76 E+00	1	0.03

Figure S1: The EI/MS spectra of unidentified compounds that are statistically differentially expressed between groups.

79.0@6.0:10

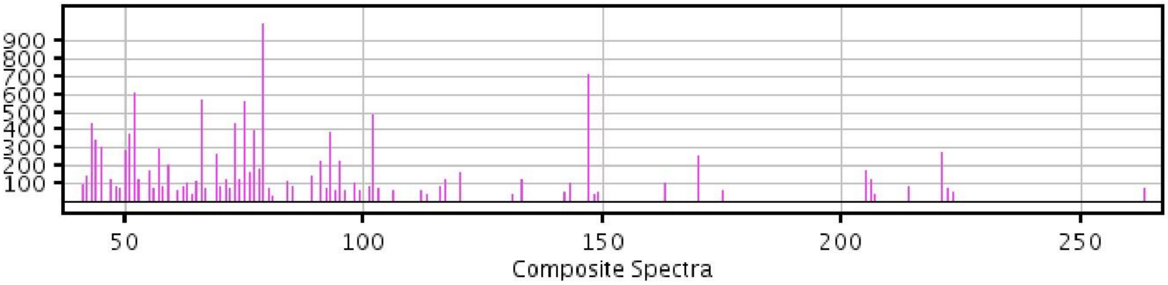

73.0@20.799995

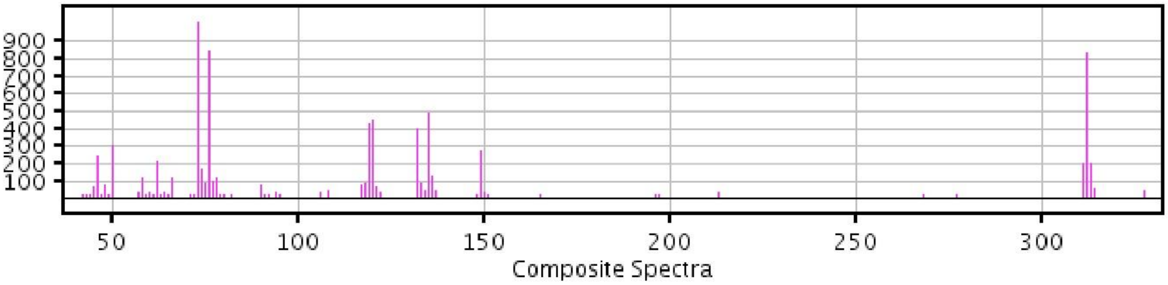

77.0@19.5

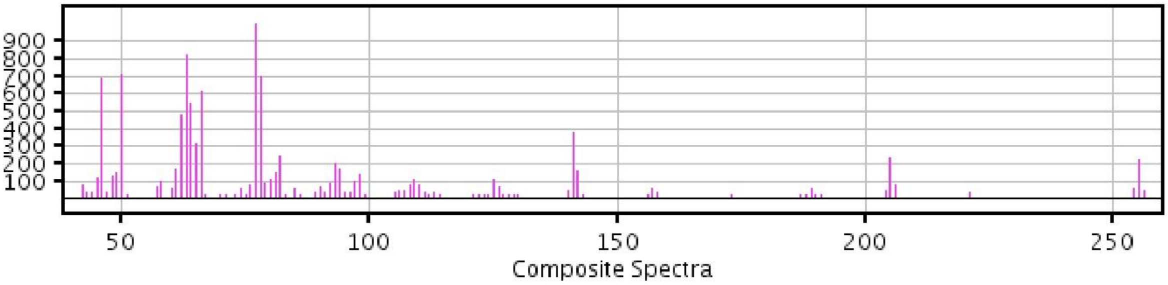

77.0@19.200006

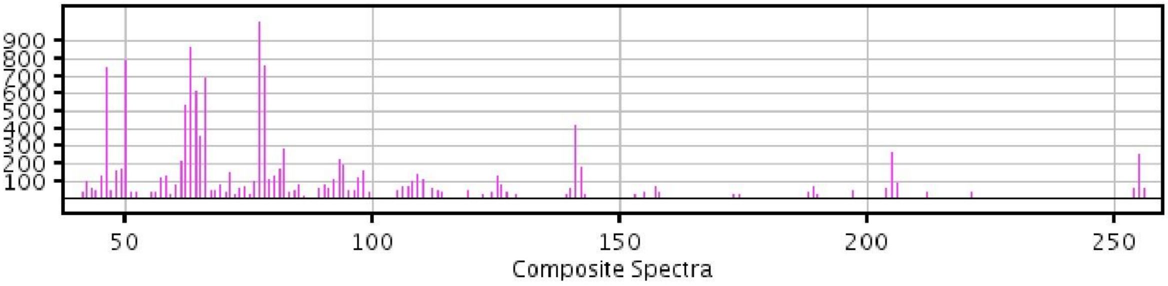

147.0@6.300001

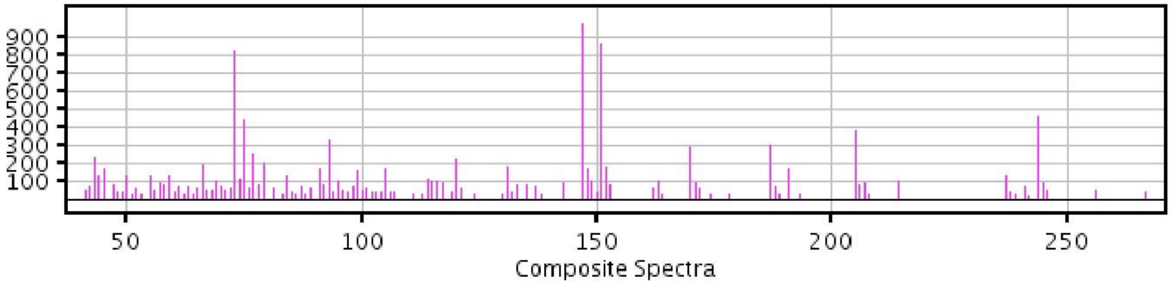

281.0@7.1:2

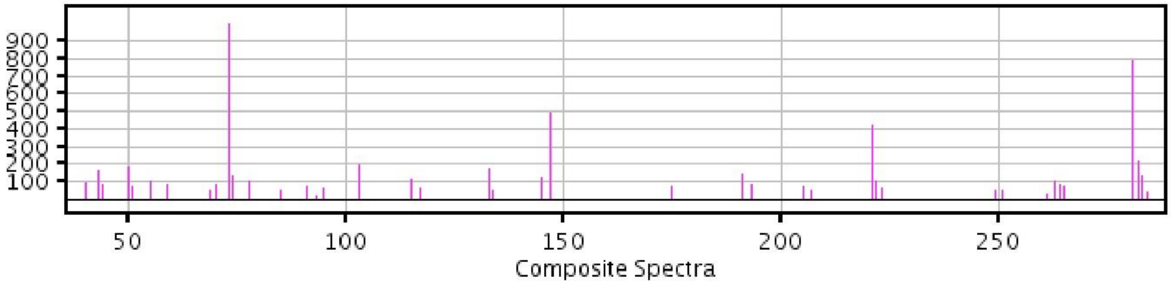

73.0@25.0:2

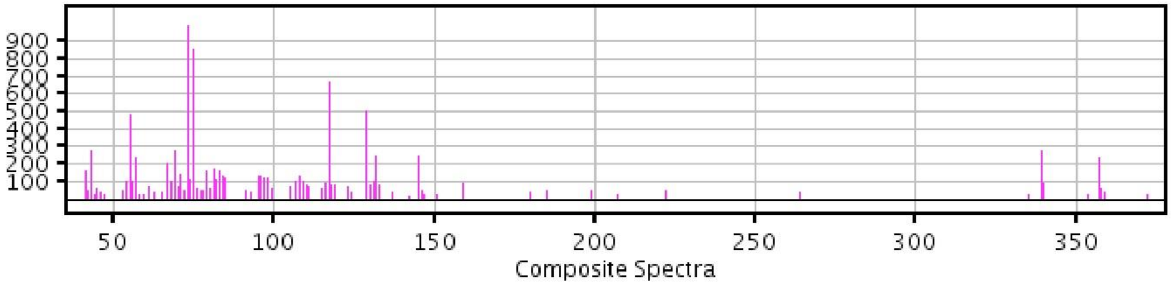

71.0@13.599994

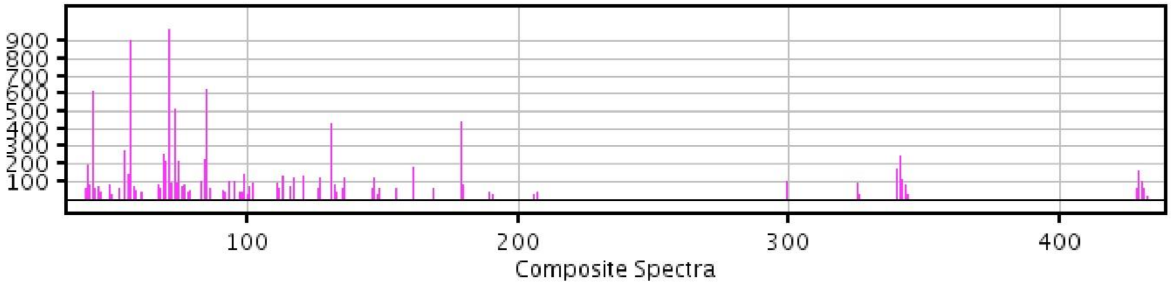

57.0@11.700005

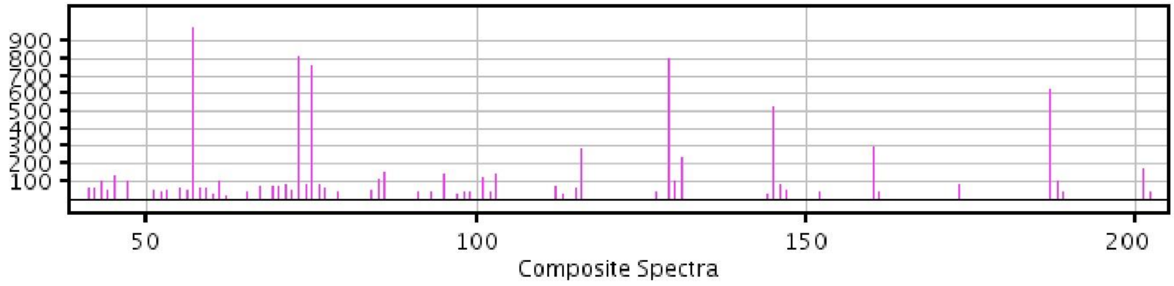

179.0@13.699998

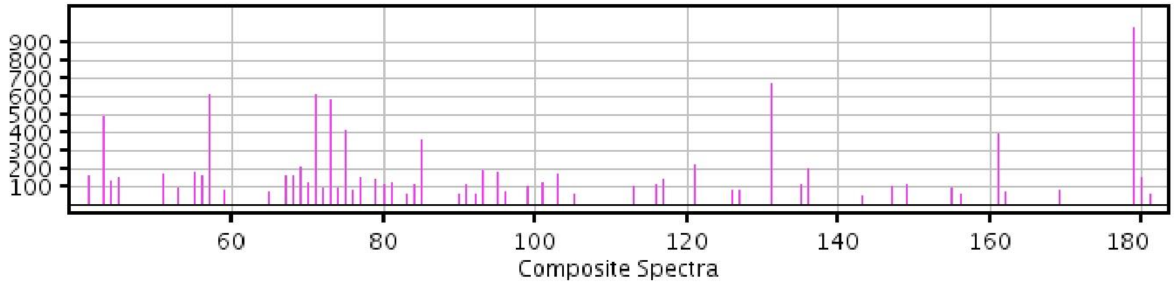

73.0@15.8

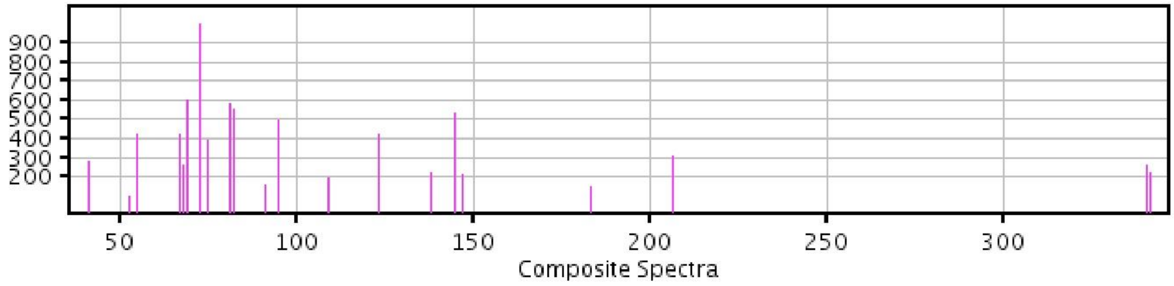

43.0@11.2

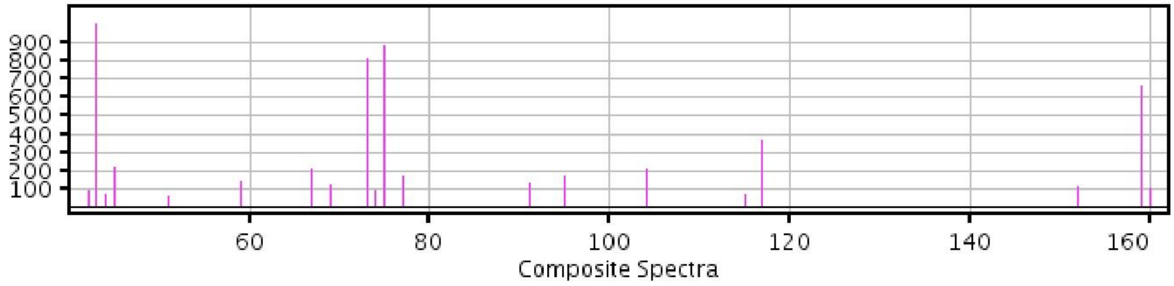

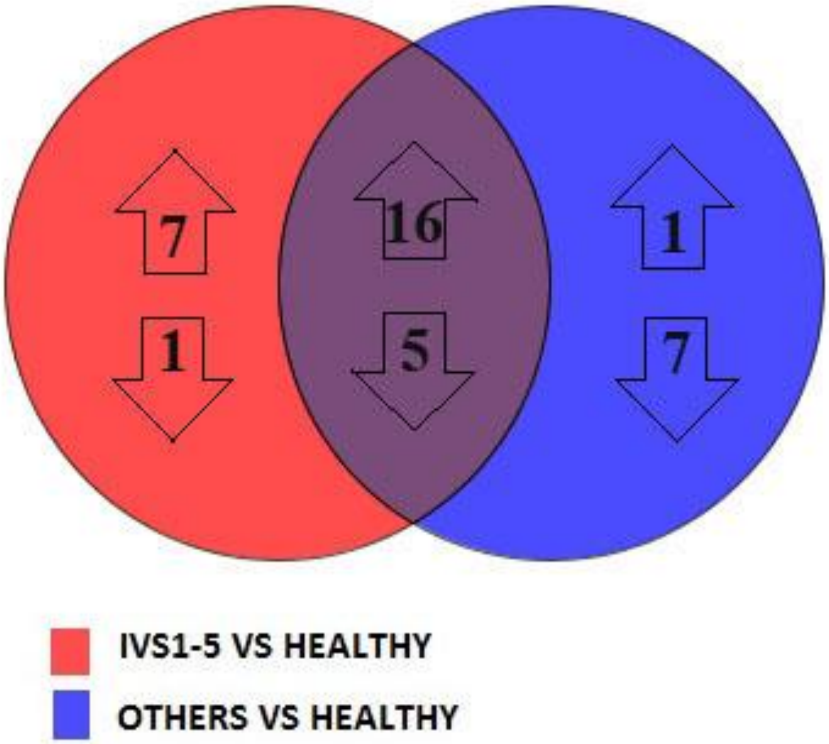

Figure S2: Venn diagrams showing the overlapping of statistically differentiating metabolites of IVS1-5 mutation with other types of mutations in comparison to healthy controls. Arrows indicating the change in metabolite as compared to controls.

Figure S3: Pathways generated by the list of metabolites discriminating between β-thalassemia patients before and after treatment with hydroxyurea and healthy controls [37-39].

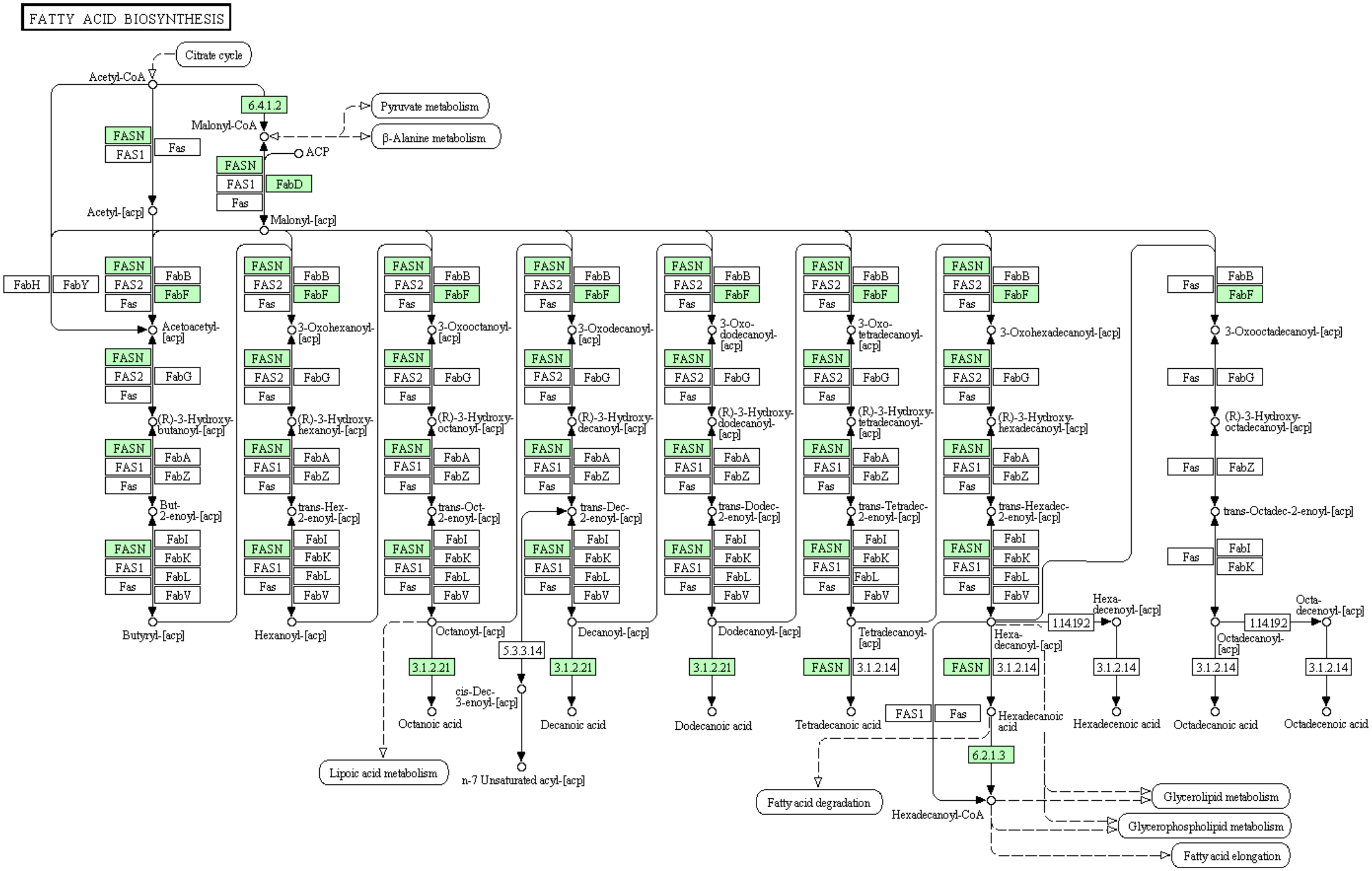

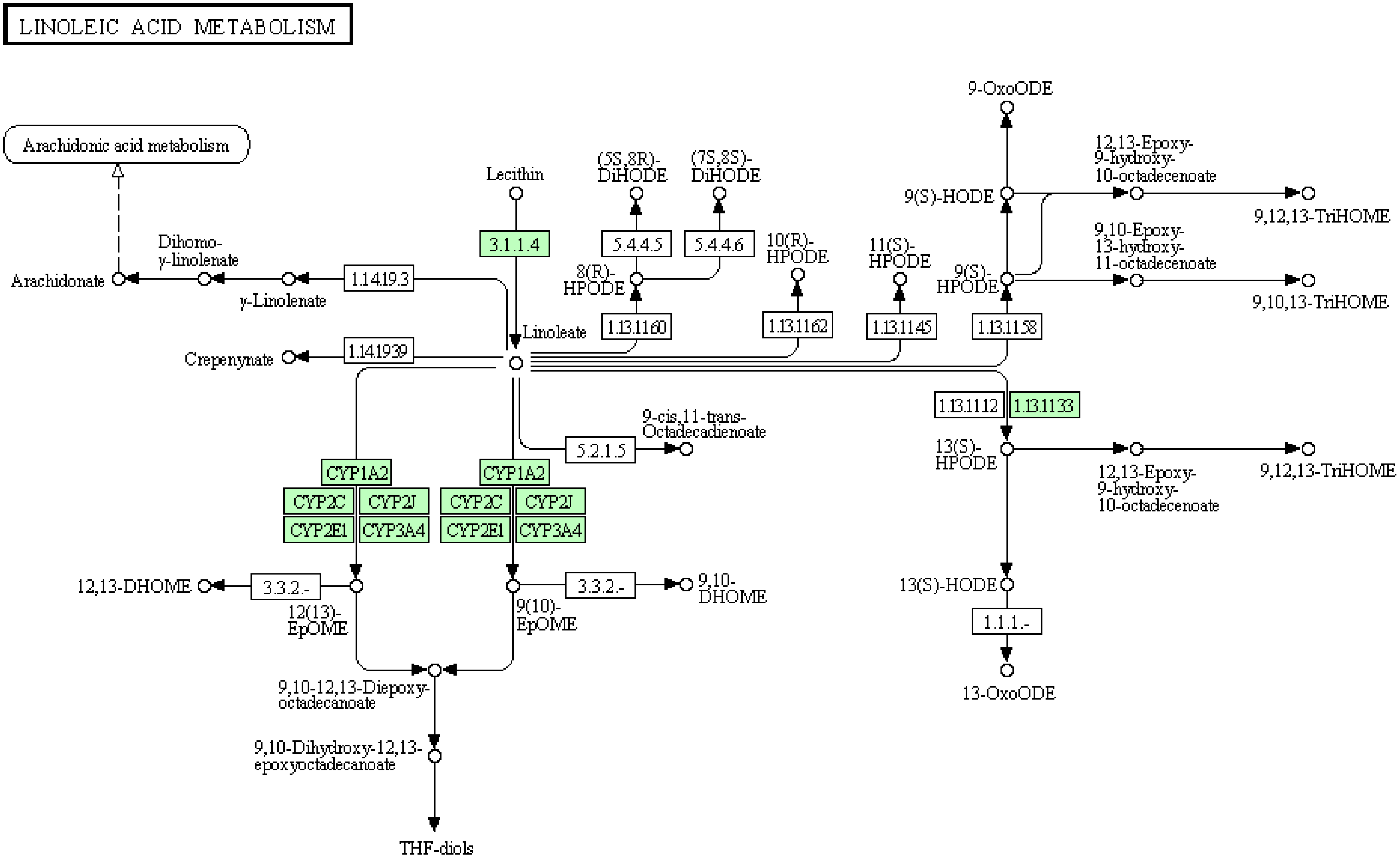
LINOLEIC ACID METABOLISM
Arachidonic acid metabolism
Arachidonate
Dihomo-
γ-linolenate
γ-Linolenate
1.14.19.3
Crepenynate
1.14.19.39
Lecithin
3.1.1.4
Linoleate
(5S,8R)-
DiHODE
(7S,8S)-
DiHODE
5.4.4.5
5.4.4.6
8(R)-
HPODE
10(R)-
HPODE
11(S)-
HPODE
1.13.11.60
1.13.11.62
1.13.11.45
1.13.11.58
9-OxoODE
9(S)-HODE
9(S)-
HPODE
12,13-Epoxy-
9-hydroxy-
10-octadecenoate
9,12,13-TriHOME
9,10-Epoxy-
13-hydroxy-
11-octadecenoate
9,10,13-TriHOME
1.13.11.12
1.13.11.33
13(S)-
HPODE
12,13-Epoxy-
9-hydroxy-
10-octadecenoate
9,12,13-TriHOME
13(S)-HODE
1.1.1.-
13-OxoODE
9-cis,11-trans-
Octadecadienoate
5.2.1.5
CYP1A2
CYP2C
CYP2J
CYP2E1
CYP3A4
CYP1A2
CYP2C
CYP2J
CYP2E1
CYP3A4
12,13-DHOME
3.3.2.-
12(13)-
EpOME
9(10)-
EpOME
3.3.2.-
9,10-
DHOME
9,10-12,13-Diepoxy-
octadecanoate
9,10-Dihydroxy-12,13-
epoxyoctadecanoate
THF-diols

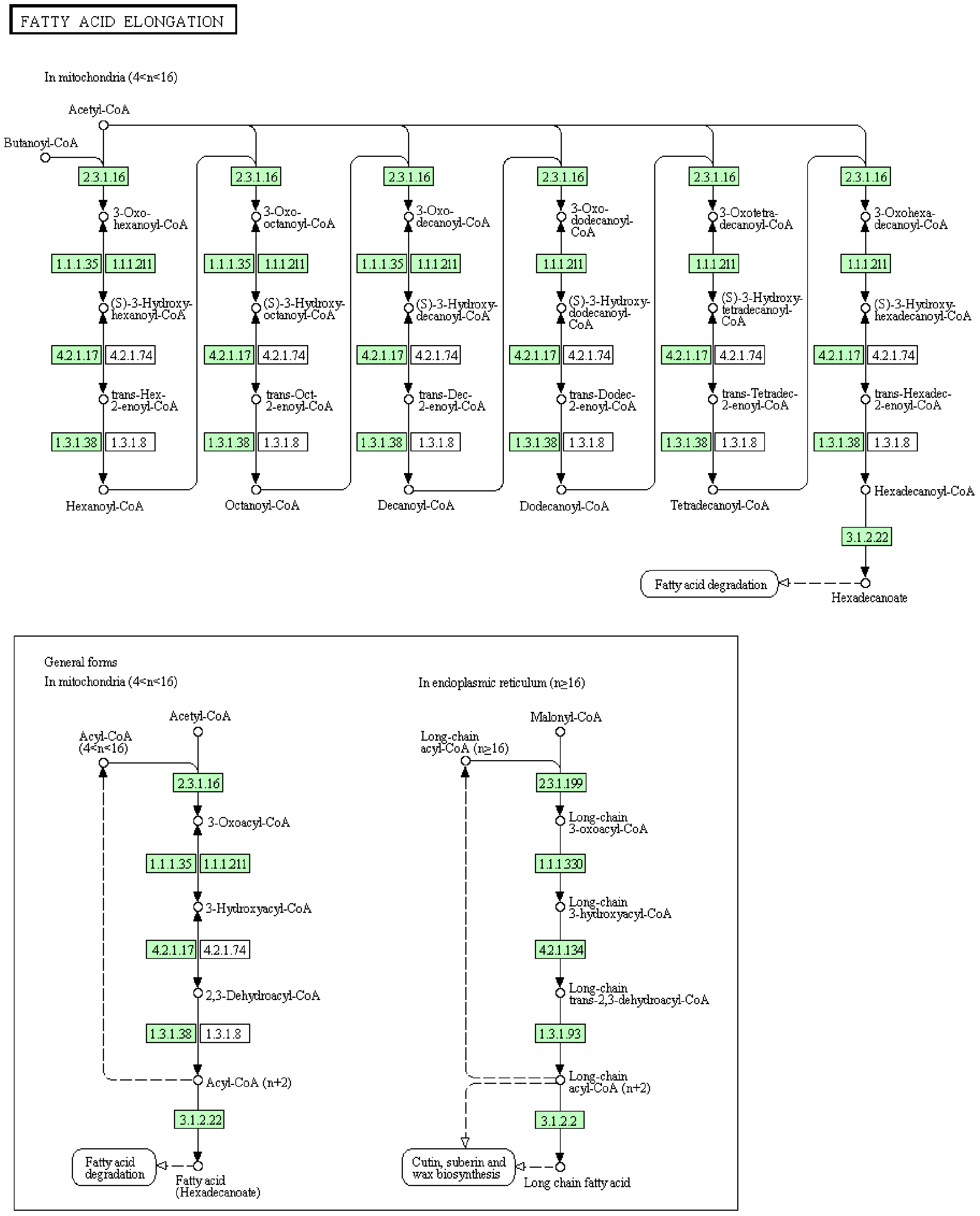
FATTY ACID ELONGATION
In mitochondria (4<n<16)
Acetyl-CoA
Butanoyl-CoA
2.3.1.16
3-Oxo-hexanoyl-CoA
3-Oxo-octanoyl-CoA
3-Oxo-decanoyl-CoA
3-Oxo-dodecanoyl-CoA
3-Oxotetra-decanoyl-CoA
3-Oxohexa-decanoyl-CoA
1.1.1.35
1.1.1.211
(S)-3-Hydroxy-hexanoyl-CoA
(S)-3-Hydroxy-octanoyl-CoA
(S)-3-Hydroxy-decanoyl-CoA
(S)-3-Hydroxy-dodecanoyl-CoA
(S)-3-Hydroxy-tetradecanoyl-CoA
(S)-3-Hydroxy-hexadecanoyl-CoA
4.2.1.17
4.2.1.74
trans-Hex-2-enoyl-CoA
trans-Oct-2-enoyl-CoA
trans-Dec-2-enoyl-CoA
trans-Dodec-2-enoyl-CoA
trans-Tetradec-2-enoyl-CoA
trans-Hexadec-2-enoyl-CoA
1.3.1.38
1.3.1.8
Hexanoyl-CoA
Octanoyl-CoA
Decanoyl-CoA
Dodecanoyl-CoA
Tetradecanoyl-CoA
Hexadecanoyl-CoA
3.1.2.22
Fatty acid degradation
Hexadecanoate
General forms
In mitochondria (4<n<16)
Acetyl-CoA
Acyl-CoA (4<n<16)
2.3.1.16
3-Oxoacyl-CoA
1.1.1.35
1.1.1.211
3-Hydroxyacyl-CoA
4.2.1.17
4.2.1.74
2,3-Dehydroacyl-CoA
1.3.1.38
1.3.1.8
Acyl-CoA (n+2)
3.1.2.22
Fatty acid degradation
Fatty acid (Hexadecanoate)
In endoplasmic reticulum (n≥16)
Malonyl-CoA
Long-chain acyl-CoA (n≥16)
2.3.1.199
Long-chain 3-oxoacyl-CoA
1.1.1.330
Long-chain 3-hydroxyacyl-CoA
4.2.1.134
Long-chain trans-2,3-dehydroacyl-CoA
1.3.1.93
Long-chain acyl-CoA (n+2)
3.1.2.2
Cutin, suberin and wax biosynthesis
Long chain fatty acid

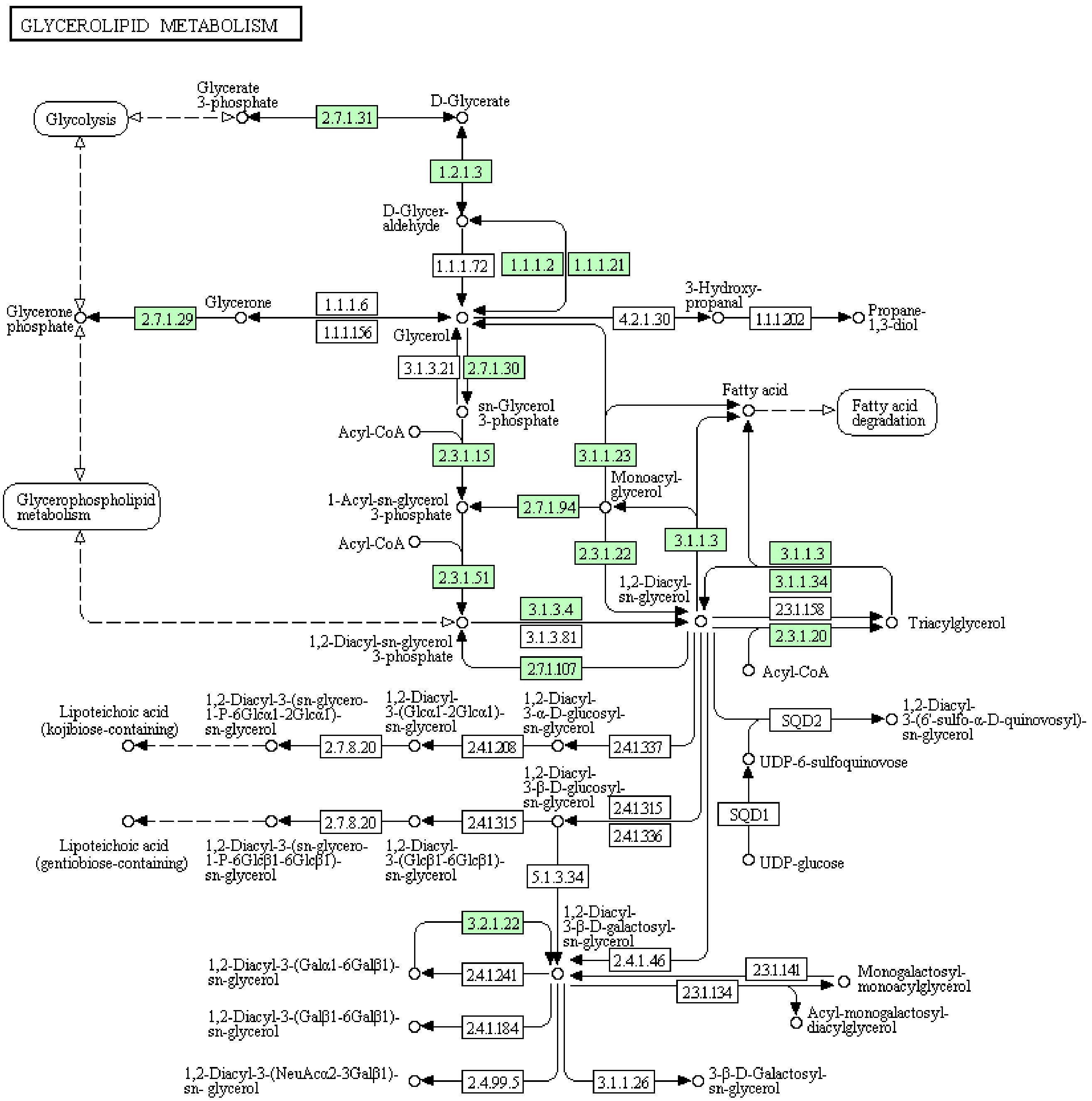
GLYCEROLIPID METABOLISM
Glycolysis
Glycerate 3-phosphate
2.7.1.31
D-Glycerate
1.2.1.3
D-Glycer-aldehyde
1.1.1.72
1.1.1.2
1.1.1.21
Glycerone phosphate
2.7.1.29
Glycerone
1.1.1.6
1.1.1.156
Glycerol
4.2.1.30
3-Hydroxy-propanal
1.1.1.202
Propane-1,3-diol
3.1.3.21
2.7.1.30
sn-Glycerol 3-phosphate
Acyl-CoA
2.3.1.15
Fatty acid
Fatty acid degradation
3.1.1.23
Glycerophospholipid metabolism
1-Acyl-sn-glycerol 3-phosphate
2.7.1.94
Monoacyl-glycerol
3.1.1.3
Acyl-CoA
2.3.1.22
2.3.1.51
1,2-Diacyl-sn-glycerol
3.1.1.3
3.1.1.34
3.1.3.4
2.3.1.158
Triacylglycerol
3.1.3.81
2.3.1.20
1,2-Diacyl-sn-glycerol 3-phosphate
2.7.1.107
Acyl-CoA
Lipoteichoic acid (kojibiose-containing)
1,2-Diacyl-3-(sn-glycero-1-P-6Glcα1-2Glcα1)-sn-glycerol
1,2-Diacyl-3-(Glcα1-2Glcα1)-sn-glycerol
1,2-Diacyl-3-α-D-glucosyl-sn-glycerol
2.7.8.20
2.4.1.208
2.4.1.337
SQD2
1,2-Diacyl-3-(6'-sulfo-α-D-quinovosyl)-sn-glycerol
UDP-6-sulfoquinovose
1,2-Diacyl-3-β-D-glucosyl-sn-glycerol
2.4.1.315
SQD1
2.7.8.20
2.4.1.315
2.4.1.336
Lipoteichoic acid (gentiobiose-containing)
1,2-Diacyl-3-(sn-glycero-1-P-6Glcβ1-6Glcβ1)-sn-glycerol
1,2-Diacyl-3-(Glcβ1-6Glcβ1)-sn-glycerol
UDP-glucose
5.1.3.34
3.2.1.22
1,2-Diacyl-3-β-D-galactosyl-sn-glycerol
2.4.1.46
1,2-Diacyl-3-(Galα1-6Galβ1)-sn-glycerol
2.4.1.241
2.3.1.141
Monogalactosyl-monoacylglycerol
2.3.1.134
Acyl-monogalactosyl-diacylglycerol
1,2-Diacyl-3-(Galβ1-6Galβ1)-sn-glycerol
2.4.1.184
1,2-Diacyl-3-(NeuAcα2-3Galβ1)-sn- glycerol
2.4.99.5
3.1.1.26
3-β-D-Galactosyl-sn-glycerol

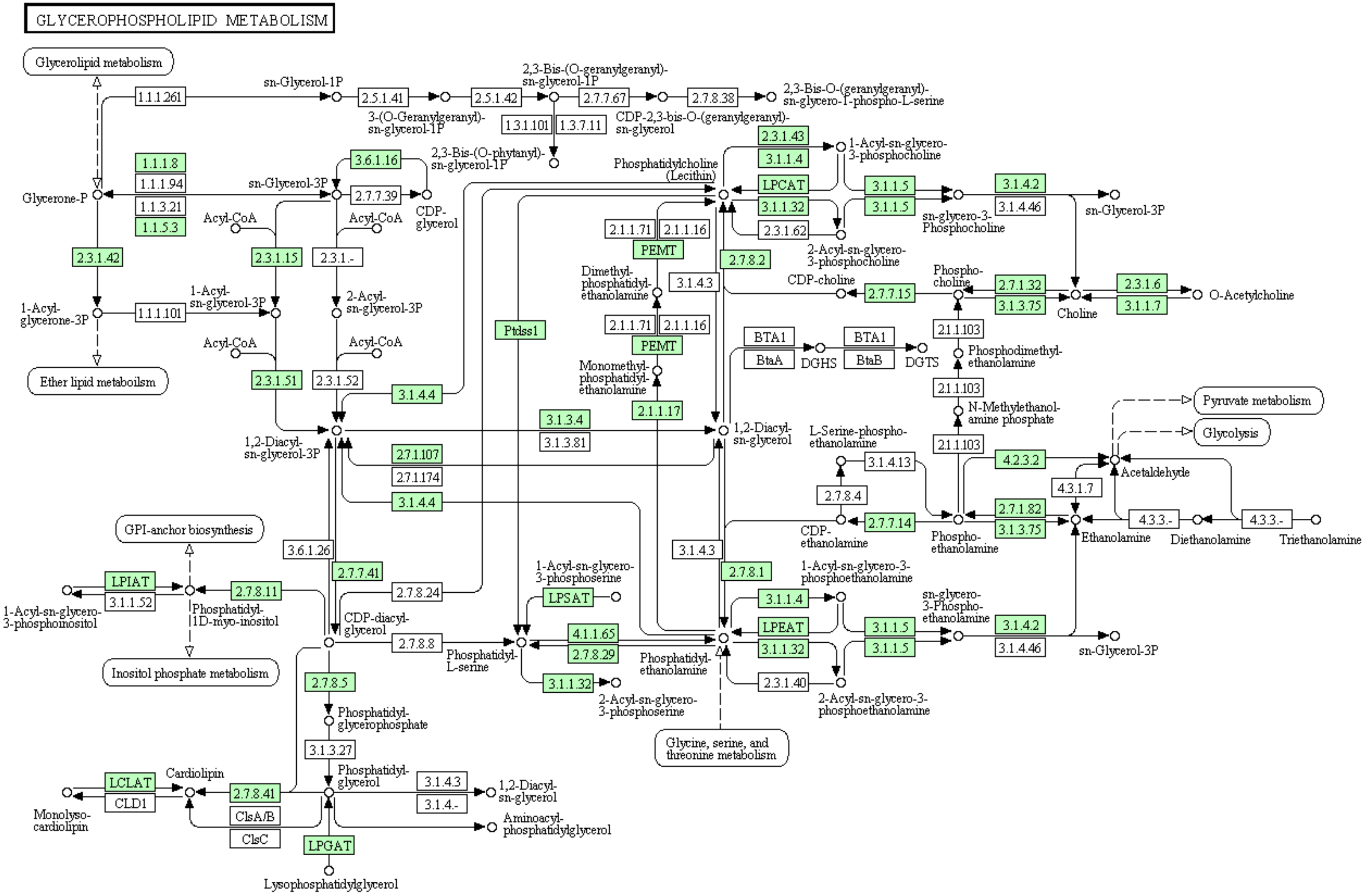
GLYCEROPHOSPHOLIPID METABOLISM
Glycerolipid metabolism
1.1.1.261
sn-Glycerol-1P
2.5.1.41
2.5.1.42
2,3-Bis-(O-geranylgeranyl)-sn-glycerol-1P
2.7.7.67
2.7.8.38
2,3-Bis-O-(geranylgeranyl)-sn-glycero-1-phospho-L-serine
3-(O-Geranylgeranyl)-sn-glycerol-1P
1.3.1.101
1.3.7.11
CDP-2,3-bis-O-(geranylgeranyl)-sn-glycerol
2,3-Bis-(O-phytanyl)-sn-glycerol-1P
1.1.1.8
1.1.1.94
1.1.3.21
1.1.5.3
Glycerone-P
sn-Glycerol-3P
3.6.1.16
2.7.7.39
CDP-glycerol
Acyl-CoA
2.3.1.42
2.3.1.15
2.3.1.-
1-Acyl-glycerone-3P
1.1.1.101
1-Acyl-sn-glycerol-3P
2-Acyl-sn-glycerol-3P
Ether lipid metaboilsm
2.3.1.51
2.3.1.52
3.1.4.4
1,2-Diacyl-sn-glycerol-3P
Phosphatidylcholine (Lecithin)
2.3.1.43
3.1.1.4
1-Acyl-sn-glycero-3-phosphocholine
LPCAT
3.1.1.32
3.1.1.5
3.1.4.2
3.1.4.46
sn-glycero-3-Phosphocholine
sn-Glycerol-3P
2.3.1.62
2-Acyl-sn-glycero-3-phosphocholine
2.1.1.71
2.1.1.16
PEMT
2.7.8.2
Dimethyl-phosphatidyl-ethanolamine
3.1.4.3
CDP-choline
2.7.7.15
Phospho-choline
2.7.1.32
3.1.3.75
Choline
2.3.1.6
3.1.1.7
O-Acetylcholine
Ptdss1
Monomethyl-phosphatidyl-ethanolamine
BTA1
BtaA
BtaB
DGHS
DGTS
2.1.1.103
Phosphodimethyl-ethanolamine
N-Methylethanol-amine phosphate
2.1.1.17
3.1.3.4
3.1.3.81
1,2-Diacyl-sn-glycerol
L-Serine-phospho-ethanolamine
3.1.4.13
Pyruvate metabolism
Glycolysis
2.7.1.107
2.7.1.174
4.2.3.2
Acetaldehyde
4.3.1.7
2.7.8.4
2.7.1.82
4.3.3.-
CDP-ethanolamine
2.7.7.14
Phospho-ethanolamine
Ethanolamine
Diethanolamine
Triethanolamine
GPI-anchor biosynthesis
3.6.1.26
2.7.7.41
2.7.8.24
2.7.8.1
1-Acyl-sn-glycero-3-phosphoserine
1-Acyl-sn-glycero-3-phosphoethanolamine
LPIAT
3.1.1.52
2.7.8.11
Phosphatidyl-1D-myo-inositol
1-Acyl-sn-glycero-3-phosphoinositol
LPSAT
LPEAT
sn-glycero-3-Phospho-ethanolamine
CDP-diacyl-glycerol
2.7.8.8
4.1.1.65
2.7.8.29
Phosphatidyl-L-serine
Phosphatidyl-ethanolamine
2.3.1.40
2-Acyl-sn-glycero-3-phosphoethanolamine
Inositol phosphate metabolism
2.7.8.5
2-Acyl-sn-glycero-3-phosphoserine
Phosphatidyl-glycerophosphate
3.1.3.27
Glycine, serine, and threonine metabolism
LCLAT
CLD1
Cardiolipin
2.7.8.41
Phosphatidyl-glycerol
3.1.4.-
Monolyso-cardiolipin
ClsA/B
ClsC
Aminoacyl-phosphatidylglycerol
LPGAT
Lysophosphatidylglycerol

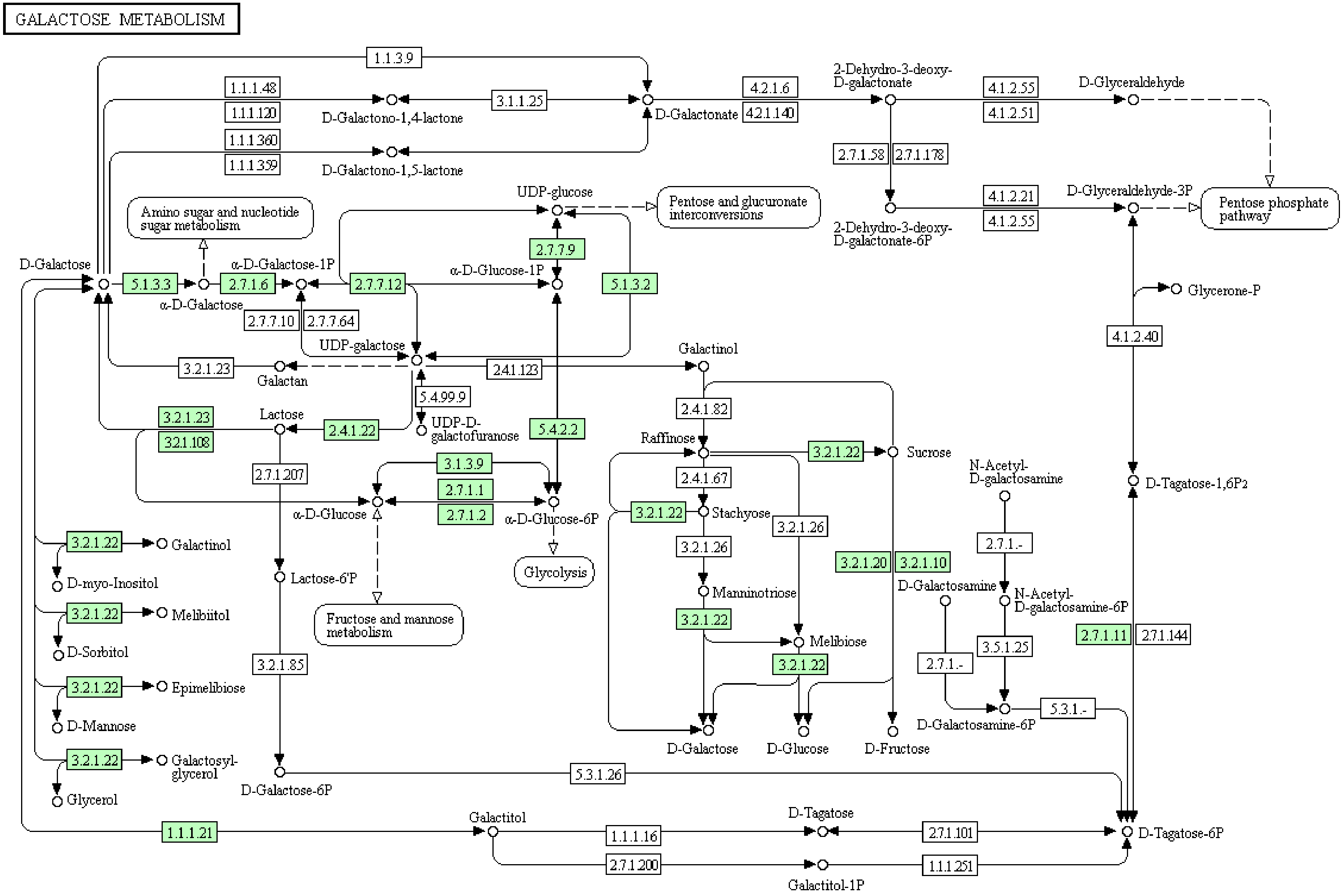
GALACTOSE METABOLISM
1.1.3.9
1.1.1.48
1.1.1.120
3.1.1.25
D-Galactono-1,4-lactone
1.1.1.360
1.1.1.359
D-Galactono-1,5-lactone
D-Galactonate
4.2.1.6
4.2.1.140
2-Dehydro-3-deoxy-D-galactonate
4.1.2.55
4.1.2.51
D-Glyceraldehyde
2.7.1.58
2.7.1.178
2-Dehydro-3-deoxy-D-galactonate-6P
4.1.2.21
4.1.2.55
D-Glyceraldehyde-3P
Pentose phosphate pathway
Amino sugar and nucleotide sugar metabolism
UDP-glucose
Pentose and glucuronate interconversions
D-Galactose
5.1.3.3
2.7.1.6
α-D-Galactose-1P
2.7.7.12
2.7.7.9
α-D-Glucose-1P
5.1.3.2
α-D-Galactose
2.7.7.10
2.7.7.64
UDP-galactose
3.2.1.23
Galactan
2.4.1.123
Galactinol
5.4.99.9
UDP-D-galactofuranose
3.2.1.23
3.2.1.108
Lactose
2.4.1.22
5.4.2.2
2.4.1.82
Raffinose
3.2.1.22
Sucrose
2.7.1.207
3.1.3.9
2.7.1.1
2.7.1.2
α-D-Glucose
α-D-Glucose-6P
2.4.1.67
3.2.1.22
Stachyose
3.2.1.26
3.2.1.26
Glycolysis
3.2.1.22
Galactinol
D-myo-Inositol
Lactose-6'P
3.2.1.22
Melibiitol
D-Sorbitol
Fructose and mannose metabolism
Manninotriose
3.2.1.22
Melibiose
3.2.1.22
3.2.1.85
3.2.1.22
Epimelibiose
D-Mannose
3.2.1.22
Galactosyl-glycerol
Glycerol
D-Galactose-6P
5.3.1.26
D-Galactose
D-Glucose
D-Fructose
3.2.1.20
3.2.1.10
N-Acetyl-D-galactosamine
2.7.1.-
D-Galactosamine
N-Acetyl-D-galactosamine-6P
3.5.1.25
2.7.1.-
D-Galactosamine-6P
5.3.1.-
Glycerone-P
4.1.2.40
D-Tagatose-1,6P2
2.7.1.11
2.7.1.144
1.1.1.21
Galactitol
1.1.1.16
D-Tagatose
2.7.1.101
D-Tagatose-6P
2.7.1.200
Galactitol-1P
1.1.1.251

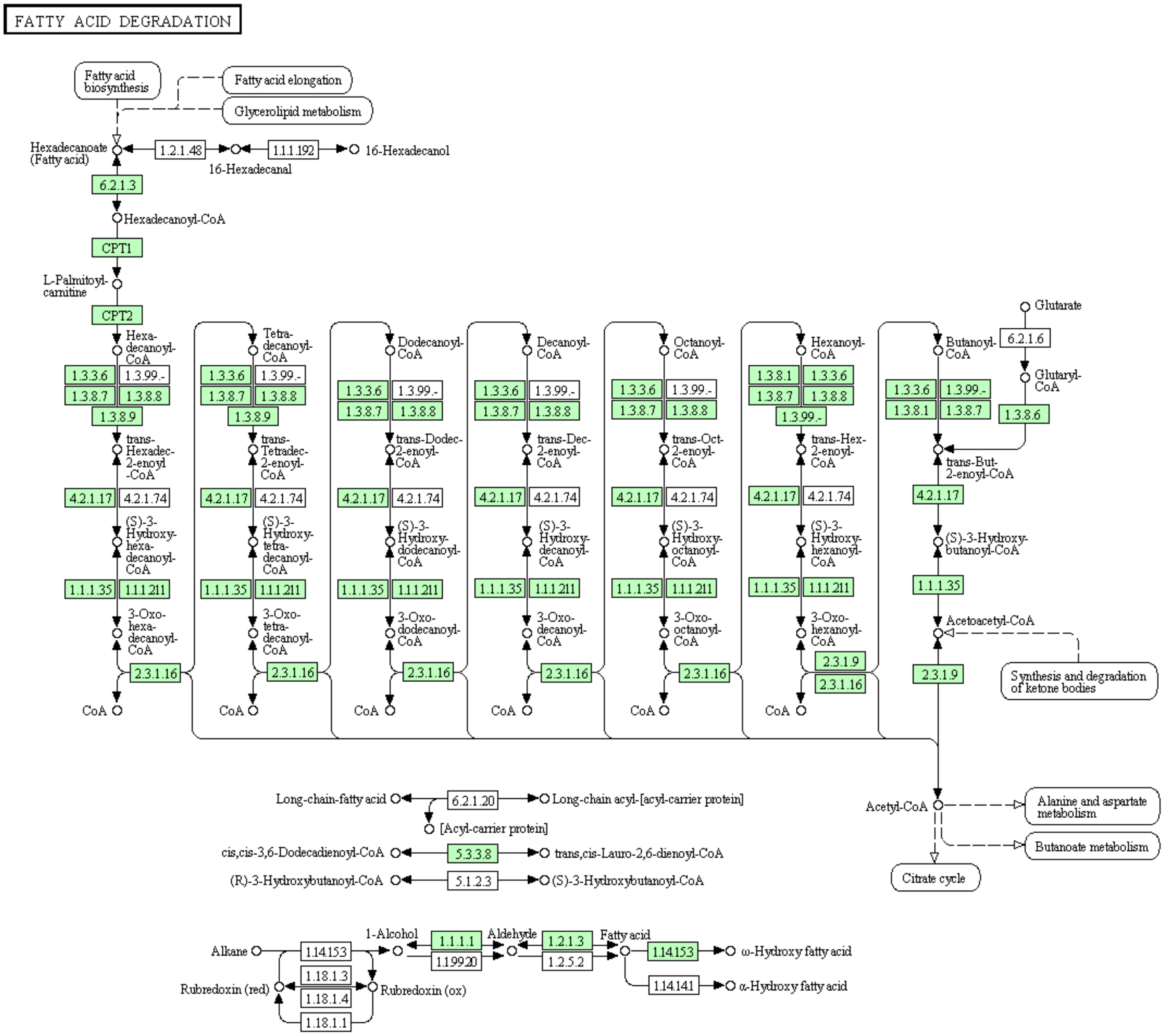

FATTY ACID DEGRADATION
Fatty acid biosynthesis
Fatty acid elongation
Glycerolipid metabolism
Hexadecanoate (Fatty acid)
1.2.1.48
1.1.1.192
16-Hexadecanol
16-Hexadecanal
6.2.1.3
Hexadecanoyl-CoA
CPT1
L-Palmitoyl-carnitine
CPT2
Hexa-decanoyl-CoA
Tetra-decanoyl-CoA
Dodecanoyl-CoA
Decanoyl-CoA
Octanoyl-CoA
Hexanoyl-CoA
Butanoyl-CoA
Glutarate
6.2.1.6
Glutaryl-CoA
1.3.8.6
1.3.3.6
1.3.99.-
1.3.8.7
1.3.8.8
1.3.8.9
1.3.8.1
trans-Hexadec-2-enoyl-CoA
trans-Tetradec-2-enoyl-CoA
trans-Dodec-2-enoyl-CoA
trans-Dec-2-enoyl-CoA
trans-Oct-2-enoyl-CoA
trans-Hex-2-enoyl-CoA
trans-But-2-enoyl-CoA
4.2.1.17
4.2.1.74
(S)-3-Hydroxy-hexa-decanoyl-CoA
(S)-3-Hydroxy-tetra-decanoyl-CoA
(S)-3-Hydroxy-dodecanoyl-CoA
(S)-3-Hydroxy-decanoyl-CoA
(S)-3-Hydroxy-octanoyl-CoA
(S)-3-Hydroxy-hexanoyl-CoA
(S)-3-Hydroxy-butanoyl-CoA
1.1.1.35
1.1.1.211
3-Oxo-hexa-decanoyl-CoA
3-Oxo-tetra-decanoyl-CoA
3-Oxo-dodecanoyl-CoA
3-Oxo-decanoyl-CoA
3-Oxo-octanoyl-CoA
3-Oxo-hexanoyl-CoA
Acetoacetyl-CoA
2.3.1.16
2.3.1.9
CoA
Synthesis and degradation of ketone bodies
Acetyl-CoA
Alanine and aspartate metabolism
Butanoate metabolism
Citrate cycle
Long-chain-fatty acid
6.2.1.20
Long-chain acyl-[acyl-carrier protein]
[Acyl-carrier protein]
cis,cis-3,6-Dodecadienoyl-CoA
5.3.3.8
trans,cis-Lauro-2,6-dienoyl-CoA
(R)-3-Hydroxybutanoyl-CoA
5.1.2.3
(S)-3-Hydroxybutanoyl-CoA
Alkane
1.14.15.3
1.18.1.3
1.18.1.4
1.18.1.1
Rubredoxin (red)
Rubredoxin (ox)
1-Alcohol
1.1.1.1
1.1.99.20
Aldehyde
1.2.1.3
1.2.5.2
Fatty acid
1.14.15.3
ω-Hydroxy fatty acid
1.14.14.1
α-Hydroxy fatty acid
